# Random Integer Lattice Generation via the Hermite Normal Form

**DOI:** 10.3390/e23111509

**Published:** 2021-11-14

**Authors:** Gengran Hu, Lin You, Liang Li, Liqin Hu, Hui Wang

**Affiliations:** 1 School of Cyberspace, Hangzhou Dianzi University, Hangzhou 310018, China; mryoulin@gmail.com (L.Y.); liangli@hdu.edu.cn (L.L.); huliqin@hdu.edu.cn (L.H.); h.wang@hdu.edu.cn (H.W.); 2 State Key Laboratory of Information Security, Institute of Information Engineering, Chinese Academy of Sciences, Beijing 100093, China

**Keywords:** random integer lattice, Hermite normal form, generation algorithm

## Abstract

Lattices used in cryptography are integer lattices. Defining and generating a “random integer lattice” are interesting topics. A generation algorithm for a random integer lattice can be used to serve as a random input of all the lattice algorithms. In this paper, we recall the definition of the random integer lattice given by G. Hu et al. and present an improved generation algorithm for it via the Hermite normal form. It can be proven that with probability ≥0.99, this algorithm outputs an *n*-dim random integer lattice within $O(n^{2})$ operations.

## 1. Introduction

Lattices are discrete subgroups in $R^{n}$. Since Ajtai’s discovery of the average-case/worst-case connection in lattice problems [1], lattice-based cryptography has attracted much attention [2,3,4,5]. Up to now, lattice-based cryptographic schemes have been considered to be a promising alternative to more traditional ones based on the factoring and discrete logarithm problems since lattice-based schemes can be resistant to efficient quantum algorithms [6]. Lattice algorithms such as LLL [7] and BKZ [8,9] are commonly used in analyzing these lattice-based schemes’ security. The lattices used in cryptography and lattice algorithms are integer lattices (discrete subgroups of $Z^{n}$). Thus, the problem of suitably defining and generating a random integer lattice is a meaningful topic. In [10], P. Q. Nguyen found that for dimensions up to 50, LLL almost outputs the shortest lattice vector, while in theory, LLL’s output is just an approximately short vector. Once we are able to generate a random integer lattice, such a generation algorithm can be used to serve as a random input for all lattice algorithms to obtain their output qualities on average.

In [1], M. Ajtai defined a family of “random integer lattices” in terms of the worst-case to average-case connection and showed how to generate one from this lattice family. For uniform $A\in Z_{q}^{n\times m}$, the lattice family is defined as $\Lambda^{⊥}\left(A\right)=\left\{\mathrm{Ax}\in Z^{m}:\mathrm{Ax}=0\in Z_{q}^{n}\right\}$. In [10], P. Q. Nguyen and D. Stehle gave a definition of the “random integer lattice” in the sense of the Haar measure, which was approximated by the Goldstein–Mayer method [11]. For large number *N*, this “random integer lattice” is uniformly chosen from the set of all n×n Hermite normal forms with the determinant equal to *N*. When *N* is prime, to generate such a random integer lattice, one only needs to set $h_{nn}=N$, $h_{in}\in \left[0,N\right)$ uniformly and $h_{ii}=1$ for i<n. This type of “random integer lattice” is used in many cryptographic applications. From the perspective of mathematics, studying whether the requirement that *N* be a prime can be removed is also a meaningful issue.

In [12], G. Maze studied the probabilistic distribution of the random HNF with a special diagonal structure, where the randomness was derived from a random square matrix whose elements were all chosen uniformly from [−B,B] for large enough *B*. In [13], G. Hu et al. introduced a different definition of randomness, in which the definition “random integer lattice” means the lattice’s HNF is chosen uniformly from all n×n HNFs whose determinants are upper bounded by a large number *M*. In the same paper [13], G. Hu et al. also presented a complete random integer lattice generation algorithm. In this algorithm, the first step is to generate a determinant. To make the final output uniform, it is necessary to compute the total number of HNFs with fixed determinant *N*. Since the total number can be figured out only in the case that the factorization of *N* is known, a subroutine to factor integers is necessary in this algorithm. In this paper, we improved this algorithm with the help of the diagonal elements’ distribution in the random HNF. This improved algorithm first generates the diagonal elements $h_{11},\cdots ,h_{n-1,n-1}$ without computing the total number of HNFs with a fixed determinant, then it uses the reverse sampling method to generate the final diagonal element $h_{nn}$. Thus, the factorization subroutine is no longer needed in this improved algorithm, which makes it more efficient.

The remainder of the paper is organized as follows. In Section 2, we give some necessary preliminaries. In Section 3, we recall the definition of the random integer lattice given by G. Hu et al. and discuss the distribution of all the diagonal elements in the random integer lattice’s HNF. For the next section, we present our improved algorithm to generate the random integer lattice via the HNF. Finally, we give our conclusion in Section 5.

## 2. Preliminaries

We denote by Z the integer ring and R the real number field. We use $GL_{n}\left(Z\right)$ to denote the general linear group over Z. For convenience, we denote the set of all n×n nonsingular integer matrices by $GL_{n}\left(R\right)\cap Z^{n\times n}$.

### Lattice and the HNF

Given a matrix $B=\left(b_{ij}\right)\in R^{n\times m}$ with rank *n*, the lattice L(B) spanned by the rows of *B* is:

$$L\left(B\right)=\left\{xB=\sum_{i=1}^{n}x_{i}b_{i}|x_{i}\in Z\right\},$$

where $b_{i}$ is the *i*-th row of *B*. We call *m* the dimension of L(B) and *n* its rank. The determinant of L(B), say det(L(B)), is defined as $\sqrt{\det(B^{T}B)}$. It is easy to see that when *B* is full-rank (n=m), its determinant becomes |det(B)|.

Two lattices $L(B_{1})$ and $L(B_{2})$ are exactly the same when there exists a matrix $U\in GL_{n}\left(Z\right)$ s.t. $B_{1}=UB_{2}$. Lattices used in cryptography are usually “integer lattices”, whose basis matrices are over Z instead of R. Thus, the space of all full-rank integer lattices is actually $\left(GL_{n}\left(R\right)\cap Z^{n\times n}\right)/GL_{n}\left(Z\right)$.

The Hermite Normal Form (HNF) is a useful tool to study integer matrices:

**Definition** **1.** *A square nonsingular integer matrix $H\in Z^{n\times n}$ is called in the HNF if:*

*• H is upper triangular, i.e., $h_{ij}=0$ for all i>j;*

*• All diagonal elements are positive, i.e., $h_{ii}>0$ for all i;*

*• All nondiagonal elements are reduced modulo the corresponding diagonal element at the same column, i.e., $0\leq h_{ij}<h_{jj}$ for all i<j.*

There exists a famous result for the HNF [14] (Chapter 2, page 66):

**Theorem** **1.** *For every $A\in GL_{n}\left(R\right)\cap Z^{n\times n}$, there exists a unique n×n matrix $B\in S_{n,Z}$ (HNF) of the form B=UA with $U\in GL_{n}\left(Z\right)$.*

By this theorem, an integer lattice corresponds to its unique HNF, implying that generating an integer lattice is actually equivalent to generating an HNF.

## 3. Random Integer Lattice

### 3.1. Definition

In this part, we refer to [13] to recall some results related to the random integer lattice.

First, for $M,N\in Z^{+}$,

$$H_{n}^{\leq }\left(M\right)≜\left\{H\;\mathrm{is}\;n-\dim\;\mathrm{HNF}|\det\left(H\right)\leq M\right\},$$

$$H_{n}\left(N\right)≜\left\{H\;\mathrm{is}\;n-\dim\;\mathrm{HNF}|\det\left(H\right)=N\right\}.$$

Gruber [15] counted the size of $|H_{n}\left(N\right)|$:

**Theorem** **2.** *If N has prime decomposition $N=p_{1}^{r_{1}}\cdots p_{t}^{r_{t}}$, then:*

$$|H_{n}\left(N\right)|=\prod_{i=1}^{t}\prod_{j=1}^{n-1}\frac{p_{i}^{r_{i}+j}-1}{p_{i}^{j}-1}.$$

There exists an asymptotic estimation for $|H_{n}^{\leq }\left(M\right)|$ in [13]:

**Theorem** **3.** *For large positive integer M,*

$$|H_{n}^{\leq }\left(M\right)|=\frac{\prod_{s=2}^{n}\zeta \left(s\right)}{n}M^{n}+O\left(M^{n-1}\log M\right).$$

*H* is called an *n*-dim random nonsingular HNF if for large integer M>0, *H* is chosen from $H_{n}^{\leq }\left(M\right)$ uniformly, and the lattice L(H) generated by such an *H* is called a random integer lattice.

### 3.2. Diagonal Distribution

In [13], Hu et al. studied the expectation and variance of every entry and the probability distribution of every diagonal entry:

**Theorem** **4.** *Let $H=(h_{ij})$ be an n-dim random nonsingular HNF with the determinant bounded by M>0 and t be an integer in [1,n−1], given an increasing subset $\left\{i_{1},\cdots ,i_{t}\right\}$ of {1,⋯,n} and its increasing complementary subset $\left\{j_{1},\cdots ,j_{n-t}\right\}$, for positive integers $b_{1}\cdots b_{t}$; when M→+∞, we have:*

(1) $$P\left(h_{i_{k},i_{k}}=b_{k}\;\mathrm{for}\;\mathrm{all}\;k\right)=\left\{\begin{array}{cc} 0 & \left(i_{t}=n\right)\\ \frac{\prod_{k=1}^{n-t-1}\zeta \left(n+1-j_{k}\right)}{\prod_{s=2}^{n}\zeta \left(s\right)}\prod_{l=1}^{t}b_{l}^{i_{l}-n-1} & \left(i_{t}<n\right) \end{array}\right.$$

If we take t=1, a one-element set T={i}(i∈[1,n−1]), and positive integers *b*, then the increasing complementary subset of *T* in {1,2,⋯,n} is {1,⋯,i−1,i+1,⋯,n}. We apply the above theorem and obtain the following corollary:

**Corollary** **1.** *Let $H=(h_{ij})$ be an n-dim random nonsingular HNF with the determinant bounded by M>0, then for i∈[1,n−1] and positive integer b, when M→+∞, we have:*

$$P\left(h_{ii}=b\right)=\frac{1}{\zeta \left(n+1-i\right)\cdot b^{n+1-i}}\;\left(b=1,2,\cdots \right).$$

*We denote this distribution of $h_{ii}$ by D(n,i).*

**Remark** **1.** *Notice that in Theorem 4, when $i_{t}<n$ and M→∞, both cases: t=1 and 1<t<n are valid conditions, which corresponds to the joint distribution of $h_{i_{k},i_{k}}\left(k=1,\cdots ,t\right)$ for 1<t<n or a marginal distribution of the single variable $h_{i_{1},i_{1}}$ for t=1 as in Corollary 1. Considering Theorem 4 and Corollary 1, it can be deduced that when M→∞, the first n−1 diagonal elements $h_{11},\cdots ,h_{n-1,n-1}$ are independent variables.*

## 4. Generating the Random Integer Lattice via the HNF

In this section, we present our random integer lattice generation algorithm via the HNF. Firstly, we introduce the inverse sampling method in probability theory to generate all the diagonal elements. Then, we generate all the nondiagonal elements accordingly.

### 4.1. Inverse Sampling Method

Given a distribution D over some ordered set *A*, we can use the inverse sampling method to generate a random variable according to the distribution D. We present two versions of the inverse sampling method: continuous-ISM and discrete-ISM.

**Theorem 5.**   (Continuous-ISM) *For distribution D over interval [a,b] with cumulative distribution function $F_{X}\left(x\right)$, choose a random y uniformly from [0,1] and compute z s.t. F(z)=y, then the resulting variable Z has distribution D.*

**Proof.**  Our goal is to prove *Z* has $F_{X}$ as its cumulative distribution function. Namely, for any x∈[a,b], we have to prove $P\left(Z\leq x\right)=F_{X}\left(x\right)$. Since *F* is a monotonically increasing function, we have:

$$P\left(Z\leq x\right)=P\left(F_{Z}\left(z\right)\leq F_{X}\left(x\right)\right)=P\left(y\leq F_{X}\left(x\right)\right)=F_{X}\left(x\right)$$

where the second equality comes from F(z)=y and the last one is a direct result of *y*’s uniformity in [0,1]. Thus, the cumulative distribution function of *Z* is actually $F_{X}$, which completes the proof.    □

**Theorem 6.**  (Discrete-ISM) *For distribution D over finite-ordered set $A={\left\{a_{k}\right\}}_{k=1}^{n}\subseteq Z$ with corresponding density $f_{k}=P\left(X=a_{k}\right)$, choose a random number y uniformly from [0,1] and compute the minimum j s.t. $\sum_{k=1}^{j}f_{k}\geq y$; then, we let $Z=a_{j}$, and Z will have distribution D.*

**Proof.**  For any $a_{j}\in A$, we need to prove $P\left(Z=a_{j}\right)=f_{j}$. Since *j* is the minimum value s.t. $\sum_{k=1}^{j}f_{k}\geq y$, we know that $\sum_{k=1}^{j-1}f_{k}<y$. Then, we have:

$$\begin{array}{cc} P(Z=a_{j}) & =P(\sum_{k=1}^{j}f_{i}\geq y,\sum_{k=1}^{j-1}f_{i}<y)\\ \; & =P\left(\sum_{k=1}^{j}f_{k}\geq y\right)-P\left(\sum_{k=1}^{j-1}f_{k}\geq y\right))\\ \; & =\sum_{k=1}^{j}f_{k}-\sum_{k=1}^{j-1}f_{k}\;(\mathrm{since}\;y\;\mathrm{is}\;\mathrm{uniform}\;\mathrm{in}\;\left[0,1\right])\\ \; & =f_{j} \end{array}$$

which completes the proof.    □

### 4.2. Generating the Random Integer Lattice via the HNF

From Section 3.1, we can generate a random integer lattice by equivalently generating a random nonsingular HNF. To begin with, we generate the first n−1 diagonal elements $h_{11},h_{22},\cdots ,h_{n-1,n-1}$. Then, we generate the last diagonal element $h_{nn}$. Finally, all the nondiagonal elements are generated, and we output the matrix *H* as a lattice basis for our random integer lattice.

#### 4.2.1. Generating $h_{11},h_{22},\cdots ,h_{n-1,n-1}$

From Corollary 1, we know that for an *n*-dim nonsingular HNF, when i∈[1,n−1], the distribution of $h_{ii}$ is:

(2) $$D\left(n,i\right):P\left(X=x\right)=\frac{1}{\zeta (n+1-i)}\cdot x^{-(n+1-i)}\;\left(x=1,2\cdots \right).$$

Therefore, we generate these diagonal elements $h_{11},h_{22},\cdots ,h_{n-1,n-1}$ according to D(n,i) by discrete-ISM (Theorem 6).

For i∈[1,n−1], we choose *y* uniformly randomly from [0,1] and increasingly iterate $j_{i}$ starting from 1 until it satisfies $\frac{1}{\zeta (n+1-i)}\sum_{k=1}^{j_{i}}k^{-(n+1-i)}\geq y$. Then, we set $h_{ii}=j_{i}$. By Theorem 6, each diagonal $h_{ii}$ has distribution D(n,i), which is what we need.

#### 4.2.2. Generating $h_{nn}$

After generating the first n−1 diagonal elements $h_{ii}$, we set $D_{n-1}≜\prod_{i=1}^{n-1}h_{ii}$. Since the determinant upper bound is *M*, the last diagonal element $h_{nn}$ should be in $\left[1,\left\lfloor \frac{M}{D_{n-1}}\right\rfloor \right]$. We point out that $D_{n-1}$ is a small number compared to *M* with high probability. More specifically, the following theorem can be proven.

**Theorem** **7.** *Let $H=(h_{ij})$ be an n-dim random nonsingular HNF with the determinant bounded by M>0; for $D_{n-1}≜\prod_{i=1}^{n-1}h_{ii}$, we have:*

$$E\left(D_{n-1}\right)=\frac{1}{\zeta (n)}\log M+O\left(1\right).$$

*Moreover, by Markov’s inequality, we find that:*

$$P\left(D_{n-1}\geq {\left(\log M\right)}^{2}\right)\leq \frac{1}{\log M}.$$

To prove Theorem 7, the following lemma from [13] is needed.

**Lemma** **1.** *Given an integer n≥4 and a large integer M>0, for any non-negative increasing sequence ${\left(s_{i}\right)}_{1\leq i\leq n}$ s.t. $s_{n}-s_{n-3}\geq 2,s_{n}-s_{n-2}\geq 1$ and a respective summation:*

$$S\left(M,s_{1}\cdots s_{n}\right)≜\sum_{a_{i}\in Z^{+},\prod_{i=1}^{n}a_{i}\leq M}a_{1}^{s_{1}}\cdots a_{n}^{s_{n}},$$

*we have the following Table 1 on asymptotic formulas for $S(M,s_{1}\cdots s_{n})$.*

*where $\zeta \left(s\right)=\sum_{i=1}^{\infty }i^{-s}$ is the well-known Riemann zeta function and the constant in the O notation is only relevant to n.*

Now, we start to prove Theorem 7.

**Proof.**  For the expectation of $D_{n-1}=\prod_{i=1}^{n-1}h_{ii}$, we find that:

$$\begin{array}{cc} E(D_{n-1}) & =\sum_{k\leq M}k\cdot P\left(D_{n-1}=k\right)\\ \; & =\frac{\sum_{k\leq M}k\cdot \left|H_{n-1}\left(k\right)\right|\cdot \sum_{a_{n}\leq M/k}a_{n}^{n-1}}{|H_{n}^{\leq }\left(M\right)|}\\ \; & =\frac{\sum_{k\leq M}\prod_{j=1}^{n-1}a_{j}\sum_{\prod_{j=1}^{n-1}a_{j}=k}\prod_{j=1}^{n-1}a_{j}^{j-1}\sum_{a_{n}\leq M/k}a_{n}^{n-1}}{|H_{n}^{\leq }\left(M\right)|}\\ \; & =\frac{\sum_{k\leq M}\sum_{\prod_{j=1}^{n-1}a_{j}=k}\prod_{j=1}^{n-1}a_{j}^{j}\sum_{a_{n}\leq M/k}a_{n}^{n-1}}{|H_{n}^{\leq }\left(M\right)|}\\ \; & =\frac{\sum_{\prod_{j=1}^{n}a_{j}\leq M}\prod_{j=1}^{n-1}a_{j}^{j}\cdot a_{n}^{n-1}}{\sum_{\prod_{j=1}^{n}a_{j}\leq M}\prod_{j=1}^{n}a_{j}^{j-1}}\\ \; & =\frac{S(M,1,2,\cdots ,n-2,n-1,n-1)}{S(M,0,1,\cdots ,n-2,n-1)}\left(\mathrm{as}\;\mathrm{in}\;\mathrm{Lemma}\;1\right)\\ \; & =\frac{\frac{\prod_{s=2}^{n-1}\zeta \left(s\right)}{n}\cdot M^{n}\log M+O\left(M^{n}\right)}{\frac{\prod_{s=2}^{n-1}\zeta \left(s\right)}{n}M^{n}+O\left(M^{n-1}\log M\right)}\left(\mathrm{by}\;\mathrm{Lemma}\;1\right)\\ \; & =\frac{\frac{\prod_{s=2}^{n-1}\zeta \left(s\right)}{n}\cdot \log M+O\left(1\right)}{\frac{\prod_{s=2}^{n-1}\zeta \left(s\right)}{n}+O\left(\log M/M\right)}\\ \; & =\frac{1}{\zeta (n)}\log M+O\left(1\right), \end{array}$$

which completes the first part of Theorem 7.

For the second part, recall that for any non-negative random variable *X*, Markov’s inequality tells us that:

P(X≥a)≤E(X)/a.

Since $D_{n-1}$ is non-negative, we apply Markov’s inequality to it by setting $a={\left(\log M\right)}^{2}$ and obtain:

$$P\left(D_{n-1}\geq {\left(\log M\right)}^{2}\right)\leq \left(\frac{1}{\zeta (n)}\log M+O\left(1\right)\right)/{\left(\log M\right)}^{2}\leq \frac{1}{\log M}$$

which completes the second part of the proof.    □

From Theorem 7, we know that $D_{n-1}$ is small compared to *M* with high probability; thus, $\left\lfloor \frac{M}{D_{n-1}}\right\rfloor$ is still large enough for us to obtain a similar result for $h_{nn}$. We think this is a relatively reasonable way to describe the distribution of $h_{nn}$. Thus, for the random nonsingular HNF with the determinant bounded by *M*, on the condition that $\prod_{i=1}^{n-1}h_{ii}=D_{n-1}$, the distribution of $h_{nn}$ is the following:

(3) $$\begin{array}{cc} \tilde{D}\left(n,M,D_{n-1}\right):\;\; & P(X=x)\\ \; & =\frac{1}{\sum_{k=1}^{\left\lfloor M/D_{n-1}\right\rfloor }k^{n-1}}\cdot x^{n-1}\\ \; & =\frac{1}{\frac{1}{n}{\left\lfloor \frac{M}{D_{n-1}}\right\rfloor }^{n}+O\left({\left\lfloor \frac{M}{D_{n-1}}\right\rfloor }^{n-1}\right)}\cdot x^{n-1}\;\left(x=1,2\cdots ,\left\lfloor \frac{M}{D_{n-1}}\right\rfloor \right). \end{array}$$

Moreover, the corresponding cumulative distribution function is:

(4) $$\begin{array}{cc} F_{X}\left(x\right) & =P(X\leq x)\\ \; & =\frac{1}{\frac{1}{n}{\left\lfloor \frac{M}{D_{n-1}}\right\rfloor }^{n}+O\left({\left\lfloor \frac{M}{D_{n-1}}\right\rfloor }^{n-1}\right)}\cdot \sum_{k=1}^{x}k^{n-1}\\ \; & =\frac{\frac{1}{n}x^{n}+O\left(x^{n-1}\right)}{\frac{1}{n}{\left\lfloor \frac{M}{D_{n-1}}\right\rfloor }^{n}+O\left({\left\lfloor \frac{M}{D_{n-1}}\right\rfloor }^{n-1}\right)}\;\left(x=1,2\cdots ,\left\lfloor \frac{M}{D_{n-1}}\right\rfloor \right). \end{array}$$

Since $\left\lfloor \frac{M}{D_{n-1}}\right\rfloor$ is still super large, we know that:

$$F_{X}\left(x\right)\approx \frac{x^{n}/n}{{\left\lfloor M/D_{n-1}\right\rfloor }^{n}/n}={\left(\frac{x}{\left\lfloor M/D_{n-1}\right\rfloor }\right)}^{n}≜G_{X}\left(x\right).$$

As a result, $G_{X}\left(x\right)$ is a rather good estimation for $F_{X}\left(x\right)$. In fact, if we define the distribution ${\tilde{D}}_{0}\left(n,M,D_{n-1}\right)$ by the cumulative distribution function $G_{X}\left(x\right)$ as follows:

(5) $$\begin{array}{cc} \tilde{D_{0}}\left(n,M,D_{n-1}\right):\;\; & P(X\leq x)\\ \; & ={\left(\frac{x}{\left\lfloor M/D_{n-1}\right\rfloor }\right)}^{n}\;\left(x=1,2\cdots ,\left\lfloor \frac{M}{D_{n-1}}\right\rfloor \right), \end{array}$$

then we have the following theorem.

**Theorem** **8.** *For large enough $M\in Z^{+}$ and positive integer $D_{n-1}=o\left(M\right)$, the statistical distance between $\tilde{D}\left(n,M,D_{n-1}\right)$ and ${\tilde{D}}_{0}\left(n,M,D_{n-1}\right)$ is at most $n\cdot O(\frac{D_{n-1}}{M})$.*

**Proof.**  According to (4), the cumulative distribution function of $\tilde{D}\left(n,M,D_{n-1}\right)$ is $F_{X}\left(x\right)=\frac{\frac{1}{n}x^{n}+O\left(x^{n-1}\right)}{\frac{1}{n}{\left\lfloor \frac{M}{D_{n-1}}\right\rfloor }^{n}+O\left({\left\lfloor \frac{M}{D_{n-1}}\right\rfloor }^{n-1}\right)}$, since the cumulative distribution function of ${\tilde{D}}_{0}\left(n,M,D_{n-1}\right)$ is $G_{X}\left(x\right)={\left(\frac{x}{\left\lfloor M/D_{n-1}\right\rfloor }\right)}^{n}$; denote $\left\lfloor \frac{M}{D_{n-1}}\right\rfloor$ by $\tilde{M}$, then $x\leq \tilde{M}$, and for every $x\in [1,\tilde{M}]$, we have:

$$\begin{array}{cc} \; & |F_{X}\left(x\right)-G_{X}\left(x\right)|\\ = & |\frac{\frac{1}{n}x^{n}+O\left(x^{n-1}\right)}{\frac{1}{n}{\tilde{M}}^{n}+O\left({\tilde{M}}^{n-1}\right)}-{\left(\frac{x}{\tilde{M}}\right)}^{n}|\\ = & |\frac{x^{n}+n\cdot O\left(x^{n-1}\right)}{{\tilde{M}}^{n}+n\cdot O\left({\tilde{M}}^{n-1}\right)}-{\left(\frac{x}{\tilde{M}}\right)}^{n}|\\ = & |\frac{\left(x^{n}+n\cdot O\left(x^{n-1}\right)\right){\tilde{M}}^{n}-\left({\tilde{M}}^{n}+n\cdot O\left({\tilde{M}}^{n-1}\right)\right)x^{n}}{{\tilde{M}}^{2n}+n\cdot O\left({\tilde{M}}^{2n-1}\right)}|\\ = & |\frac{n\cdot O\left(x^{n-1}\right){\tilde{M}}^{n}-n\cdot O\left({\tilde{M}}^{n-1}\right)x^{n}}{{\tilde{M}}^{2n}+n\cdot O\left({\tilde{M}}^{2n-1}\right)}|\\ = & |\frac{n\cdot O\left({\tilde{M}}^{n-1}\right){\tilde{M}}^{n}-n\cdot O\left({\tilde{M}}^{n-1}\right){\tilde{M}}^{n}}{{\tilde{M}}^{2n}+n\cdot O\left({\tilde{M}}^{2n-1}\right)}|\left(\sin\mathrm{ce}\;x\leq \tilde{M}\right)\\ = & |\frac{n\cdot O({\tilde{M}}^{2n-1})}{{\tilde{M}}^{2n}+n\cdot O\left({\tilde{M}}^{2n-1}\right)}|\\ = & |\frac{n\cdot O(\frac{1}{\tilde{M}})}{1+n\cdot O(\frac{1}{\tilde{M}})}|=n\cdot O\left(\frac{1}{\tilde{M}}\right)=n\cdot O\left(\frac{D_{n-1}}{M}\right) \end{array}$$

which implies that the statistical distances $\tilde{D}\left(n,M,D_{n-1}\right)$ and ${\tilde{D}}_{0}\left(n,M,D_{n-1}\right)$ are bounded by $n\cdot O(\frac{D_{n-1}}{M})$.    □

Since $\left\lfloor M/D_{n-1}\right\rfloor$ is still super large, we can generate $h_{nn}$ according to ${\tilde{D}}_{0}\left(n,M,D_{n-1}\right)$ (close enough to $\tilde{D}\left(n,M,D_{n-1}\right)$) by continuous-ISM (Theorem 5).

We choose *y* uniformly randomly from [0,1] and compute $z\in R^{+}$ s.t.:

$${\left(\frac{z}{\left\lfloor M/D_{n-1}\right\rfloor }\right)}^{n}=y.$$

Then, we set $h_{nn}=\left\lfloor z\right\rceil$. By Theorems 6 and 8, the diagonal $h_{nn}$ has distribution ${\tilde{D}}_{0}\left(n,M,D_{n-1}\right)$, which is close enough to $\tilde{D}\left(n,M,D_{n-1}\right)$.

#### 4.2.3. Generating $h_{ij}\left(i\neq j\right)$

This part is relatively easier. For i,j=1,⋯,n, let $h_{ij}$ be chosen from $\left[0,h_{jj}\right)$ uniformly randomly if i<j and let $h_{ij}=0$ if i>j.

#### 4.2.4. Correctness

By the discussion above, for large enough M>0, the distribution of the diagonal $h_{11},\cdots ,h_{nn}$ generated by this algorithm is close enough to its distribution as a random nonsingular HNF. For i<j∈[1,n], since a random nonsingular HNF’s $h_{ij}$ is uniform in $\left[0,h_{jj}\right)$ and $h_{ij}$ is generated in the same way, we know that the output of this algorithm is also close enough to a real random nonsingular HNF, which implies the correctness of this algorithm.

### 4.3. Algorithm 1: Generate Random Integer Lattice

Now we present the Algorithm 1 to generate a random integer lattice.
**Algorithm 1:** Random Integer Lattice Generation**Require:** Dimension *n*, large integer *M***Ensure:** *n*-dim random integer lattice L with det(L)≤M **Step 1:** Generate $h_{11},\cdots ,h_{n-1,n-1}$
 $D_{0}=1$
 **for**
i=1 to n−1 **do**

     $j_{i}=1$, $s_{i}=1$

     choose $y_{i}\in \left[0,1\right]$ uniformly

     **while** $s_{i}<\zeta \left(n+1-i\right)\cdot y_{i}$ **do**

         $j_{i}=j_{i}+1$

         $s_{i}=s_{i}+j_{i}^{-(n+1-i)}$

     **end while**

     $D_{i}=D_{i-1}\cdot j_{i}$

     set $h_{ii}=j_{i}$

 **end for**

 **Step 2:** Generate $h_{nn}$

 choose y∈[0,1] uniformly

 $z=y^{1/n}$

 $z=z\cdot \lfloor \frac{M}{D_{n-1}}\rfloor$

 set $h_{nn}=\left\lfloor z\right\rceil$

 **Step 3:** Generate $h_{ij}\left(i\neq j\right)$

 **for**
j=1 to *n* **do**

     **for** i=1 to j−1 **do**

         choose $h_{ij}\in \left[0,h_{jj}\right)$ uniformly

     **end for**

     **for** i=j+1 to *n* **do**

         set $h_{ij}=0$

     **end for**

 **end for**

 **Step 4:** Set $H=(h_{ij})$, and output L(H)


### 4.4. Time Complexity of Algorithm 1

Now, we analyze the time complexity of Algorithm 1. Obviously, the most time-consuming part of Algorithm 1 is the floating-point operations $s_{i}=s_{i}+j_{i}^{-(n+1-i)}$ inside the while iteration for each *i* in Step 1. Denote the number of computing $s_{i}=s_{i}+j_{i}^{-(n+1-i)}$ in the *i*-th while iteration by T(i). Notice that:

$$P\left(h_{ii}=1\right)=\frac{1}{\zeta (n+1-i)};$$

since ζ(s) converges to one quite fast as *s* grows, the majority of $h_{ii}$ will be set to one. In fact, by the numerical results, we have following result:

**Fact 1**: For any integer n≥10,

$$\frac{1}{\prod_{s=10}^{n}\zeta \left(s\right)}\geq 0.999.$$

By this fact, for i≤n−10, all the $h_{ii}$ are very likely to be set to one, implying that T(1),T(2),⋯,T(n−10)=0 with probability ≥0.999. Then, we consider T(n−9),T(n−8),⋯,T(n−1). If we set the probability bound for each T(i) to be 0.999, then by accurate numerical results, we have the following Table 2:

Thus, we have the following theorem:

**Theorem** **9.** *The number of floating-point operations performed in Algorithm 1 is bounded by 1300 with probability ≥0.99.*

**Proof.**  By the above table, $\sum_{i=n-9}^{n-1}T\left(i\right)$ is bounded by 640 with probability $\geq 0.999^{9}$. Since T(1),T(2),⋯,T(n−10)=0 with probability ≥0.999, we know that $\sum_{i=1}^{n-1}T\left(i\right)$ is bounded by 640 with probability $\geq 0.999^{10}\geq 0.99$. Notice that each $s_{i}=s_{i}+j_{i}^{-(n+1-i)}$ needs two floating-point operations, and it also needs another four floating-point operations to generate $h_{nn}$ in Step 2; thus, with probability ≥0.99, the total number of floating-point operations performed in Algorithm 1 is bounded by 640×2+4=1284<1300, which completes the proof. □

**Remark** **2.** *We point out that the accuracy of the floating-point affects the actual running time of Algorithm 1. By experiments, 150 bit are a suitable option.*

It is not hard to see that in Algorithm 1, besides the floating-point operations, the remaining parts of Step 1, Step 2, and Step 3 take $O\left(n^{2}\right),O\left(1\right)$, and $O(n^{2})$ operations, respectively. Combining this with Theorem 9, we have the following result:

**Theorem** **10.** *Algorithm 1 outputs a random integer lattice within $O(n^{2})$ operations with probability ≥0.99.*

## 5. Conclusions

In this paper, we presented an improved algorithm for generating random integer lattices and discussed its time complexity. We proved that with probability ≥0.99, this algorithm outputs an *n*-dim random integer lattice within $O(n^{2})$ operations. We pointed out that there is still space for improvement of our algorithm, and we leave this as an open problem. 

## Figures and Tables

**Table 1:** Asymptotic formulas of $S(M,s_{1}\cdots s_{n})$ in different cases.

$S(M,s_{1}\cdots s_{n})$	If
$\frac{\prod_{j=1}^{n-1}\zeta \left(s_{n}+1-s_{j}\right)}{s_{n}+1}M^{s_{n}+1}+O\left(M^{s_{n}}\log M\right)$	$s_{n-3}\leq s_{n-2}<s_{n-1}<s_{n}$
$\frac{\prod_{j=1}^{n-1}\zeta \left(s_{n}+1-s_{j}\right)}{s_{n}+1}M^{s_{n}+1}+O\left(M^{s_{n}}{\left(\log M\right)}^{2}\right)$	$s_{n-3}<s_{n-2}=s_{n-1}<s_{n}$
$\frac{\prod_{j=1}^{n-2}\zeta \left(s_{n}+1-s_{j}\right)}{s_{n}+1}M^{s_{n}+1}\log M+O\left(M^{s_{n}+1}\right)$	$s_{n-3}\leq s_{n-2}<s_{n-1}=s_{n}$

**Table 2:** Upper bound for T(i) with probability≥0.999.

T(i)	Upper Bound
T(n−9)	0
T(n−8)	1
T(n−7)	1
T(n−6)	1
T(n−5)	2
T(n−4)	3
T(n−3)	6
T(n−2)	19
T(n−1)	607

## Data Availability

Not applicable.
